# Drug knowledge discovery via multi-task learning and pre-trained models

**DOI:** 10.1186/s12911-021-01614-7

**Published:** 2021-11-16

**Authors:** Dongfang Li, Ying Xiong, Baotian Hu, Buzhou Tang, Weihua Peng, Qingcai Chen

**Affiliations:** 1 Harbin Institute of Technology (Shenzhen), Shenzhen, China; 2 Peng Cheng Laboratory, Shenzhen, China; 3 Baidu, International Technology (Shenzhen) Co., Ltd, Shenzhen, China

**Keywords:** Gene mutation, Drug repurposing, Biomedical language models

## Abstract

**Background:**

Drug repurposing is to find new indications of approved drugs, which is essential for investigating new uses for approved or investigational drug efficiency. The active gene annotation corpus (named AGAC) is annotated by human experts, which was developed to support knowledge discovery for drug repurposing. The AGAC track of the BioNLP Open Shared Tasks using this corpus is organized by EMNLP-BioNLP 2019, where the “Selective annotation” attribution makes AGAC track more challenging than other traditional sequence labeling tasks. In this work, we show our methods for trigger word detection (Task 1) and its thematic role identification (Task 2) in the AGAC track. As a step forward to drug repurposing research, our work can also be applied to large-scale automatic extraction of medical text knowledge.

**Methods:**

To meet the challenges of the two tasks, we consider Task 1 as the medical name entity recognition (NER), which cultivates molecular phenomena related to gene mutation. And we regard Task 2 as a relation extraction task, which captures the thematic roles between entities. In this work, we exploit pre-trained biomedical language representation models (e.g., BioBERT) in the information extraction pipeline for mutation-disease knowledge collection from PubMed. Moreover, we design the fine-tuning framework by using a multi-task learning technique and extra features. We further investigate different approaches to consolidate and transfer the knowledge from varying sources and illustrate the performance of our model on the AGAC corpus. Our approach is based on fine-tuned BERT, BioBERT, NCBI BERT, and ClinicalBERT using multi-task learning. Further experiments show the effectiveness of knowledge transformation and the ensemble integration of models of two tasks. We conduct a performance comparison of various algorithms. We also do an ablation study on the development set of Task 1 to examine the effectiveness of each component of our method.

**Results:**

Compared with competitor methods, our model obtained the highest Precision (0.63), Recall (0.56), and F-score value (0.60) in Task 1, which ranks first place. It outperformed the baseline method provided by the organizers by 0.10 in F-score. The model shared the same encoding layers for the named entity recognition and relation extraction parts. And we obtained a second high F-score (0.25) in Task 2 with a simple but effective framework.

**Conclusions:**

Experimental results on the benchmark annotation of genes with active mutation-centric function changes corpus show that integrating pre-trained biomedical language representation models (i.e., BERT, NCBI BERT, ClinicalBERT, BioBERT) into a pipe of information extraction methods with multi-task learning can improve the ability to collect mutation-disease knowledge from PubMed.

## Background

Drug repurposing is a strategy used to identify new uses for approved or investigational drugs that are beyond the scope of the original medical indication. It focuses on predicting the effective off-label usages of existing drugs on the market. These drugs may have valid or expired licenses. Both researchers and the industry pay more attention to the repurposing usages of the drugs with expired licenses. Generally, PubMed^1^ is considered a significant source of knowledge discovery because it stores a growing number of scientific discovery reports. It requires further development of more automated methods. Recently, utilizing the natural language processing techniques to find and mine medication-related information from the text (e.g., PubMed) for drug repurposing has been a promising exploration theme [1–4].

For the objective of drug repurposing, the active gene annotation corpus (AGAC) was created as a benchmark dataset [5]. The AGAC track is the portion of the BioNLP Open Shared Task 2019 [6], which points to accumulate content mining approaches among the BioNLP community to focus on drug-oriented knowledge discovery. It comprises three assignments to extract mutation-disease information from PubMed abstracts: trigger words NER, thematic roles identification, and mutation-disease information extraction. One mission of this track is to extend the effectiveness of drug discovery. Discovering the relationship of a drug with its target mutant gene needs to consider the functional changes of the corresponding mutant gene and the drug's pharmacological activity. The gene-function change-disease knowledge in this track contains the relationship between mutation and disease and indicates the function change of the mutation, i.e., gain of function (GOF) and loss of function (LOF). To this end, we focus on the tasks of trigger words NER and thematic roles identification tasks.

The large-scale pre-trained language models have recently become the basis for various natural language processing tasks [7, 8]. They achieved remarkable performance across a wide range of tasks [9], e.g., text classification, natural language inference, question answering. One popular used pre-trained language model is BERT which is proposed by Devin et al. [7]. BERT firstly trains bidirectional transformers [10] on the unannotated large-scale corpus from the general domain, and the pre-trained model is then fine-tuned to adapt to downstream tasks. This fine-tuning process is regarded as transfer learning, where BERT acquires knowledge from the large-scale corpus and transfers it to downstream tasks. Although BERT was developed for general-purpose language understanding, there are likewise several pre-trained models that follow BERT architecture leveraging domain-specific knowledge effectively from a large set of unannotated biomedical texts (e.g., PubMed abstracts, clinical notes), such as SciBERT [11], BioBERT [12], NCBI BERT [13], Clinical BERT [14, 15]. In particular, SciBERT [11] leverages unsupervised pre-training on a large multi-domain corpus of scientific publications. BioBERT (BERT for Biomedical Text Mining) [12] further trained Google’s BERT on PubMed abstracts (4500M words). NCBI BERT (a.k.a BlueBERT) [13] was pre-trained on PubMed abstracts and clinical discharge summaries (i.e., MIMIC-III notes) [16]. ClinicalBERT [15] was clinically oriented BERT models initialized with original BERT and BioBERT parameters, and some of them pre-trained on PubMed abstracts, PMC articles, MIMIC III notes [16] and a subset of discharge summaries. Knowledge can be transferred by these models effectively from a large number of unlabeled texts to biomedical text mining models with minimum task-specific architecture revisions.

## Methods

### Pre-trained language model

The BERT model architecture is a multi-layer bidirectional Transformer encoder [10] that is based on the original self-attention mechanism. The input representation is a concatenation of WordPiece embeddings, segment embeddings and positional embeddings. A particular classification token “[CLS]” is inserted as the first token and separated token “[SEP]” is added as the last token. Given an input token sequence $x={x}_{1}, \dots , {x}_{T}$, BERT's output is $H= {h}_{1}, \dots , {h}_{T}$ after 12 stacked self-attention blocks. It is firstly pre-trained with two strategies on large-scale unlabeled text, i.e., masked language model and next sentence prediction.

The pre-trained BERT model provides a powerful context-dependent sentence representation and can be applied to various kinds of downstream tasks, i.e., machine reading comprehension and text classification, through the fine-tuning procedure. Based on the BERT architecture, several domain-specific language representation models are pre-trained on large-scale biomedical corpora (e.g., PubMed abstracts, clinical notes) for biomedical text mining. These models can be transferred effectively from many unlabeled texts to biomedical text mining models with minimal task-specific architecture modifications.

**Fig. 1:**
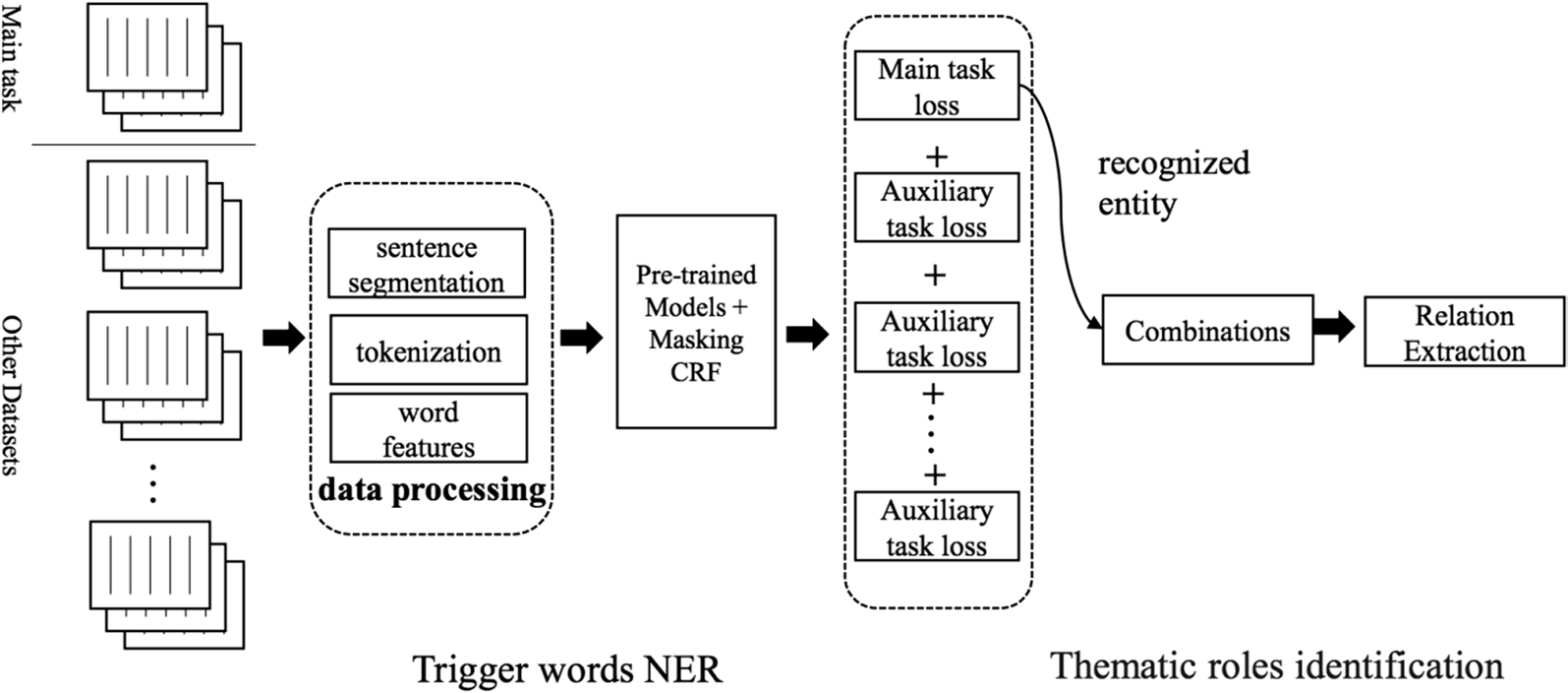
The architecture of our method. We start by splitting the PubMed abstract into sentences, tagging them as words, and extracting several features, such as POS tags. NER offsets and entity identification are then performed based on the BERT-based approach, and finally the relationship of each potential entity pair is predicted

Hence, the BERT model can easily be extended to the medical domain information extraction pipeline, first extracting the trigger words and determining the relationship between these entities, as shown in Fig. 1.

### Task 1: trigger words NER

**Fig. 2:**
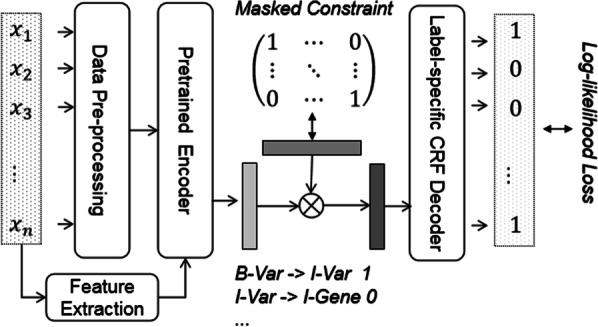
The base architecture diagram of our NER model

Task 1 aims to identify trigger words in the PubMed digest and annotate them as correct trigger markers or entities (Var, MPA, Interaction, Pathway, CPA, Reg, PosReg, NegReg, Disease, Gene, Protein, Enzyme). As shown in Fig. 2, it can be seen as an NER task involving the identification of many domain-specific proper nouns in the biomedical corpus [17, 18]. For example, the sentence is“Our results showed that SHP-2 E76K mutation caused myeloproliferative disease in mice”, and we need to extract entities: SHP-2 (Gene), E76K mutation (Var) and myeloproliferative (disease). The challenge of this task comes from two parts, unbalanced entity type distribution and selective annotation (i.e., if any necessary gene, mutation, disease mentions are missing in the sentence, other named entities that appear in the sentence will not be annotated).

We first split each PubMed abstracts into sentences using '\n' or '.', and convert each sentence into words by NLTK tokenizer.^2^ After that, words are further tokenized into sub-tokens $x={x}_{1}, \dots , {x}_{T}$. Then we use a representation based on the BERT from the last layer $H= {h}_{1}, \dots , {h}_{T}$. In order to make better use of the word-level information, POS tagging labels and word shape embedding representation [19] of each word are also concatenated into the output of BERT, passing through a single projection layer, followed by the conditional random fields (CRF) layer [20] with a masking constraint to calculate the token-level label probability $P= {p}_{1}, \dots , {p}_{T}$. If a word is tokenized into several tokens, each token will be given the same tagging labels. Transition mask with invalid moves as 0 and valid as 1.

When fine-tuning the BERT, we found that the performance of the model performed better in the case of BIO for the selection of the tagging schemes compared to BIOES. We further extend our model to multi-task learning jointly trained by sharing the architecture and parameters. Although the discrepancy in different datasets, multi-task means joint learning with other biomedical corpora. The assumption is to make more efficient use of the data and to encourage the models to learn more generalized representations. More specially, the same token-level information and BERT encoder are shared and each data set has a specific output layer, e.g., CRF layer. Our final loss function is obtained as follows:

$$-\sum {\lambda }_{{c}_{i}}logP\left({y}_{{c}_{i}}|{x}_{{c}_{i}}\right)+ {\lambda }_{r}{\Vert W\Vert }_{2}$$

where ${y}_{{c}_{i}}$ denotes true tag sequence and ${x}_{{c}_{i}}$ denotes the input tokens for corpora ${c}_{i}$, ${\lambda }_{{c}_{i}}$ and ${\lambda }_{r}$ are weighted parameters.

### Task 2: thematic roles identification

Task 2 is to identify the thematic roles (Theme of, Cause of) between trigger words. For example, the sentence is “two protein-truncating DNMs ... in SHROOM3,...”, and the relationship between the “DNMs (Var)” and the “SHROOM3 (Gene)” is “ThemeOf”. Note that the cross-sentence relations, which account for 96% of the data set, are challenges for the model to capture long dependencies.

We treat it as the multiclass classification problem by introducing “no relation (NA)” label. When constructing the training data of Task 2, we use the relational tuples of which two entities are no more than one sentence away. For NA label, random sampling is performed. In the testing process, relation label will be assigned to the corresponding thematic role when its probability is maximum and larger than the threshold. Otherwise, it will be predicted as no relation.

**Fig. 3:**
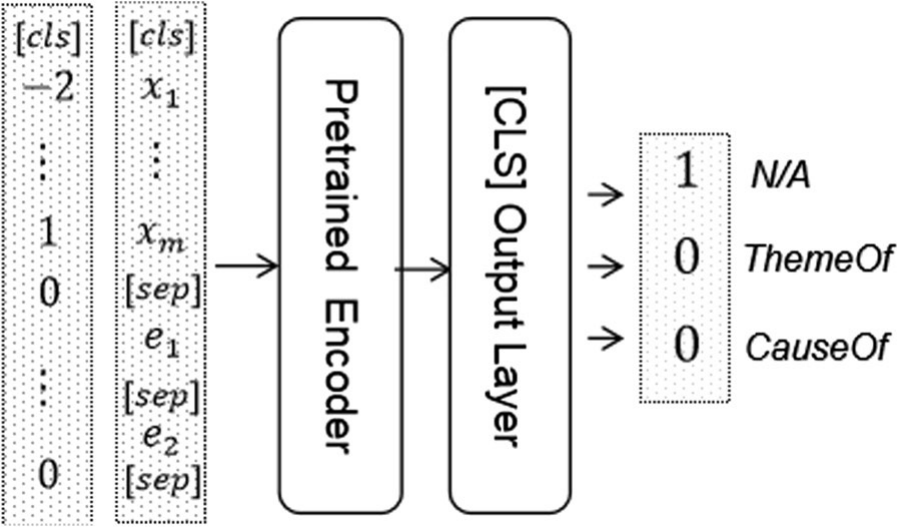
The base architecture diagram of our RE model

We also anonymously use a predefined tag (such as %Disease) to represent a target named entity. And we additionally append two concrete predicted entity words separated by the [SEP] tag after each sentence shown in Fig. 3. Following [21], we also add the token-level relative distance to the subject entity information for each token, i.e., 0 for the position *t* between two entities, *t-s* for tokens before first entity and *t-e* for tokens after second entity, where *s*, *e* are the starting and ending positions of first and second entity after tokenization, respectively. The relation logits of two entities are performed using a single output layer from the BERT as $y=softmax(W{h}_{cls}+b)$ where ${h}_{cls}$ denotes the hidden state of the first special token ([CLS]).

**Fig. 4:**
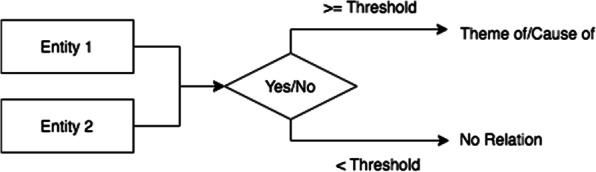
The two-stage inference procedure for task 2. Yes: there is a relation between two entities; No: no relation exists. Here we set the threshold equals 0.5

Furthermore, we notice that most pairs of entities are unrelated (i.e., NA label) that causes a large label imbalance. To alleviate the problem, similar to [22], we use a two-stage inference procedure for task 2 as shown in Fig. 4. In the first stage, the model needs to determine whether the relationship exists for a given pair of entities, i.e., binary classification (NA or REL). Random sampling and down-sampling methods are used to select the negative data. In the second stage, we learn a model trained only using relation pairs to distinguish their labels between the two corresponding entities (Theme of / Cause of). After that, for a given pair of entities at the time of testing, the model of the first stage is first applied to predict whether there is a relationship between them. If the relation label is predicted, the model of the second stage is applied to predict the thematic roles.

### Experimental setup

The AGAC track organizers develop an active gene annotation corpus (AGAC) [4, 23], for the sake of knowledge discovery in drug repurposing. The track corpus consists of 1250 PubMed abstracts: 250 for public, 1000 for final evaluation. Although the total number of abstracts is small, it contains 2534 sentences, among which 3317 named entities and 2729 relationship groups are distributed. Among them, there are 1428 named entities of the Bio-concept Named Entities type, 905 named entities of the Regulatory Named Entities type, and 984 named entities of the Other Entities type. We randomly split the public texts into train and development data sets with the radio of 8:2. The training set is used to learn model parameters, the development set to select optimal hyper-parameters. For evaluation results, we measure the trigger words recognition and thematic roles extraction performance with ${F}_{1}$ score. Table 1 shows the external data sets used under the joint learning method. The BIO form of these data sets is different from that of Task 1; hence we use different projection and CRF layers. But it is not that the more data sets, the better the model performance. We found that the NCBI disease [24] and BC5CDR [25] datasets are helpful for the final results, and the performance is reduced when using BC2GM [26] and 2010 i2b2/VA dataset [27]. We use three metrics to evaluate the performance of all methods: Precision (P), Recall (R), F-score (F1).

**Table 1:** Datasets statistics for joint learning in recognizing the trigger words

Datasets	BC5CDR	NCBI disease	BC2GM	2010 i2b2/VA
# Train	4559	5423	12,573	16,315
# Dev	4580	922	2518	–
# Test	4796	939	5037	27,626

### Experiment settings

We tried the original BERT,^3^ NCBI BERT,^4^ ClinicalBERT^5^ and BioBERT^6^ pre-trained models. Each training example is pruned to at most 384 and 512 tokens for named entity recognition (NER) and relation extraction (RE). We use a batch size of 5 for NER, and 32 for RE. We also use the hierarchical learning rate in the training process so that the pre-trained parameters and the newly added parameters converge at different optimization processes. For fine-tuning, we train the models for 20 epochs using a learning rate of $2*{10}^{-5}$ for pre-trained weights and $3*{10}^{-5}$ for others. The learning parameters were selected based on the best performance on the dev set. For trigger word detection, we ensemble 5 models from fivefold cross-validation and 2 models using the normal training-validation approach. For the identification of thematic roles, we ensemble 3 models that used all the construction data in training.

## Results

### Main results

The results of the two tasks with the pre-trained model for trigger words NER and thematic roles identification are presented in Table 2. We show a comparison of the performance of the development set results using different pre-trained models. From Table 2, we can see that the pre-trained model outperforms the classical BiLSTM + CRF labeling approach for the general domain [28]. From the last four lines of two tasks, we can see that different pre-trained models have different results for the same experimental setup. It demonstrates the validity of performing pre-training tasks in the medical or biomedical domain.

**Table 2:** Model comparison in development set with different pre-trained models

Task	Model	P	R	F_1_
Trigger words recognition	BiLSTM + CRF	0.478	0.408	0.440
	BERT_base_	0.497	0.448	0.471
	NCBI BERT	*0.553*	0.453	0.498
	ClinicalBERT	0.523	0.486	0.504
	BioBERT	0.511	*0.529*	*0.519*
Thematic roles identification	BERT_base_	0.758	0.890	0.818
	NCBI BERT	0.778	0.879	0.826
	ClinicalBERT	0.796	0.913	0.850
	BioBERT	0.807	0.891	0.847
	ClinicalBERT-TS	0.810	*0.917*	*0.860*
	BioBERT-TS	*0.813*	0.894	0.852

The models (except BiLSTM + CRF) are jointly trained by using NCBI dataset, BC5CDR dataset, and our training set. BioBERT performs better than others in Task 1, while ClinicalBERT achieves best F_1_ in Task 2. The two-step training process (i.e., TS) further improves the performance

The results for Task 1 are presented in Table 3. The baseline method of Task 1 is to use BERT to learn the semantic structure of the text and then output sequence labels. The difference in performance across labels stems partly from the unbalanced distribution of trigger labels [29]. Our method performs better than the previous best and provides a significant improvement over the previous state-of-the-art methods. Table 4 summarizes the results for Task 2. The baseline method of Task 2 is to use the traditional support vector machine to classify the relationship. Our method improves over the baseline model and multi-stage training is found to be effective for relationship extraction. However, there is a large discrepancy between the performance of our approach on the development set and the performance of the test set: one reason is that the test set may be quite different from our constructed development set; on the other hand, this also is relevant to the way we use recognized entities (e.g., sentence-level or document-level pair combinations).

**Table 3:** Comparison of Precision (P), Recall (R) and F1 scores for trigger word detection

Label	P	R	F_1_
CPA	0.39	0.27	0.32
Disease	0.57	0.57	0.57
Enzyme	0.75	0.16	0.26
Gene	0.71	0.64	0.68
Interaction	0.50	0.29	0.36
MPA	0.46	0.47	0.47
NegReg	0.71	0.62	0.66
Pathway	0.83	0.36	0.50
PosReg	0.64	0.61	0.63
Protein	0.32	0.17	0.22
Reg	0.75	0.50	0.60
Var	0.64	0.63	0.64
ALL (ours)	*0.63*	*0.56*	*0.60*
ALL (baseline)	0.50	0.51	0.50

**Table 4:** Comparison of Precision (P), Recall (R) and F1 score for prediction of thematic roles

Label	P	R	F_1_
Cause of	0.60	0.26	0.36
Theme of	0.63	0.11	0.19
ALL (ours)	0.61	0.16	0.25
ALL (baseline)	0.05	0.02	0.03

### Ablation study

To test the validity of each component of our approach, we performed ablation experiments using the development set of Task 1.

As illustrated in Table 5, we can see that adding a layer of BiLSTM behind the BERT encoder does not improve the performance of the model, resulting in an ${F}_{1}$ loss of 0.04. For the NER task, external features are likely to be an improvement in performance of the model. Therefore, we verified the validity of the lexical and POS labels on task 1 and found that adding this information makes the value of ${F}_{1}$ increase by more than 0.01. In addition, jointly learning using other datasets of named entity recognition task can also improve the results of the model.

**Table 5:** Ablation study of Task 1 in development set

Model	P	R	F_1_
BioBERT	0.511	0.529	0.519
w/ BiLSTM	0.502	0.448	0.473
w/o Word shape	0.539	0.453	0.492
w/o POS tags	0.518	0.482	0.499
w/o Multi-task learning	0.492	0.478	0.484

## Discussion

Identifying disease-related genes and their related changes is a challenging task for biomedical research. With the help of the AGAC dataset, we used fine-tuning and multi-task learning techniques to identify the trigger labels and thematic roles in PubMed abstracts. Our work can also be applied to large-scale automatic extraction of medical text knowledge, which should propel drug repurposing research.

As mentioned in the paper [29] of the task organizer, different from the traditional sequence labeling problem, there is selective partial labeling in the AGAC dataset (that is, it is labeled when the sentence fits the GOF/LOF topic). In addition, due to the complexity of labeling and the uneven distribution of medical knowledge, the distribution of AGAC data sets in some types of entities is different, and the number of abstracts for labeling is limited. The limitation of training data may affect the learning process of the model. In this paper, we use cross-validation and early stopping methods to avoid overfitting as much as possible. When dealing with NER joint learning with multiple corpora and multiple entity types, a critical issue is whether it introduces noisy labels or significantly decreases performance, e.g., a disease in NCBI corpus is labeled as DISEASE while it is not in the BC2GM corpus. In this work, we migrate the problem through different task layers. Another question is whether the performance of entities of the same type from different corpora can be compared. We argue that it is an open question whether equivalent comparisons can be made, considering differences in the entity type definition, annotation standard, and data quality.

We also conducted the error analysis. There are several types of errors: the first is the *abbreviation* problem [30], but we can use the abbreviation tool in the post-processing process to obtain its corresponding full name, for example: Cd is Cadmium, AF is Atrial fibrillation. However, this processing method will encounter a specific abbreviation corresponding to different full names in different articles, for example: AD is the abbreviation of Alzheimer's disease, but in another paragraph is the abbreviation of acute distress. The second common mistake is that the *specific gene name* is not in the vocabulary of pre-trained models, making it difficult to identify. The last kind of weakness is related to our method. We employ the pipeline to solve the tasks, with NER comes before RE. However, pipeline systems are prone to error propagation. In the field of general natural language processing, the latest work [31, 32] uses the framework of encoder-decoder to generate triples. In order to solve the problem of triplet overlap, Wei et al. [33] also propose a Hierarchical Binary Tagging method to model the relationship as a function that maps the subject in the sentence to the object. However, they only proved useful on the sentence-level dataset. For processed text data in the medical field, the entities and relationships to be extracted are often distributed in different paragraphs. Different entities need to cross sentence combinations to judge the relationship, which is also a challenge for the current model. Besides, in the AGAC corpus, long contexts also make it more challenging to model sequence information. As mentioned in [34], truncated short text segments may prevent the model from capturing long dependencies and global information in the document.

## Conclusions

In this paper, we integrated pre-trained biomedical language representation models into an information extraction pipeline to collect mutational disease knowledge from PubMed. Specially, we investigated the use of pre-trained models (i.e., BERT, NCBI BERT, ClinicalBERT, and BioBERT) to fine-tune new tasks to reduce the risk of overfitting. By considering the relationship between different data sets, we get better results. Experimental results of benchmark annotation of genes with active mutation-centric functional changes show that pre-trained models help improve the baseline to obtain state-of-the-art performance. In future work, we will explore how to simultaneously train entity recognition and relationship extraction tasks to reduce the cascading errors caused by the pipeline model in biomedical information extraction.

## Data Availability

The code and processed data during the current study are available from the corresponding author upon reasonable requests.

## Abbreviations

BERT: Bidirectional encoder representations from transformers
AGAC: Active gene annotation corpus
BioNLP: Biomedical natural language processing
NER: Named entity recognition
RE: Relation extraction
CRF: Conditional random field
NLTK: Natural language toolkit
BiLSTM: Bidirectional long short-term memory networks
GOF: Gain of function
LOF: Loss of function
MIMIC: Medical Information Mart for Intensive Care
NCBI: National Center for Biotechnology Information

## Footnotes

1 https://www.ncbi.nlm.nih.gov/pubmed/.

2 https://www.nltk.org.

3 https://github.com/google-research/bert.

4 https://github.com/ncbi-nlp/NCBI_BERT.

5 https://github.com/EmilyAlsentzer/clinicalBERT.

6 https://github.com/dmis-lab/biobert.
